# Fungal Community Composition and Diversity Vary With Soil Horizons in a Subtropical Forest

**DOI:** 10.3389/fmicb.2021.650440

**Published:** 2021-07-01

**Authors:** Xia Luo, Kezhong Liu, Yuyu Shen, Guojing Yao, Wenguang Yang, Peter E. Mortimer, Heng Gui

**Affiliations:** ^1^School of Biological Science and Food Engineering, Chuzhou University, Anhui, China; ^2^CAS Key Laboratory for Plant Diversity and Biogeography of East Asia, Kunming Institute of Botany, Chinese Academy of Sciences, Kunming, China; ^3^Centre for Mountain Futures, Kunming Institute of Botany, Chinese Academy of Sciences, Kunming, China

**Keywords:** Ascomycota, Basidiomycota, fungi, illumina sequencing, soil horizon, soil organic matter

## Abstract

Soil fungal communities, which drive many ecosystem processes, vary across soil horizons. However, how fungal communities are influenced by soil horizon layers remains largely unstudied. In this study, soil samples were collected from the organic horizon (O horizon) and mineral matter horizon (M horizon) in two sites of Dabie Mountain, China, and the effects of the two horizons on the soil fungal community composition were assessed based on Illumina MiSeq sequencing. Our results showed that soil fungal community composition varied with soil horizons, and soil fungal species richness and diversity in the O horizon were significantly higher than that in the M horizon. Total organic carbon (TOC), total organic nitrogen (TON), alkali-hydrolyzable nitrogen (AHN), available potassium (AK), and available phosphorus (AP) significantly influenced fungal community composition, abundance, and diversity across the two horizons (*P* < 0.05). Furthermore, precipitation was found to have a significant effect on fungal community composition. Our results demonstrate changes in fungal communities across soil horizons and highlight the importance of soil organic matter on fungal communities and diversity.

## Introduction

Soil microorganisms play important roles in soil ecosystem functions (van Leeuwen et al., 2017). Importantly, the diversity and structure of soil microorganisms have been regarded as important indicators of soil function and quality (Huang et al., 2018). Fungi are integral to the soil microorganisms that drive many critical ecosystem processes, including the cycling of essential elements (e.g., carbon, nitrogen, phosphorus, and sulfur) and nutrients, litter decomposition, plant growth regulation, and influence on the coexistence and diversity of plant species (Teste et al., 2017; Yang et al., 2017; Liu et al., 2018). However, we still possess limited understanding of variations in soil fungal communities across different soil microenvironments (Shi et al., 2014; He et al., 2017). In recent years, high-throughput sequencing has not only enhanced our understanding of microbial community composition in forest types but also allowed us to better detect community compositions or changes in the proportions of specific taxa inhabiting many environments (Truong et al., 2017; Tedersoo et al., 2018; Větrovský et al., 2018).

Some studies have shown that fungal diversity and community composition vary according to soil profile (van Leeuwen et al., 2017; Yang et al., 2017). Variation in soil profiles is related to variation in soil horizons. Physical, chemical, microbial, and soil properties as well as their interactions with soil organic matter vary across soil horizons (Rumpel and Kögel-Knabner, 2011). The O horizon, with its higher level of soil organic matter content and darker color, is dominated by organic soil materials such as leaves, needles, twigs, moss, or lichens in various states of decomposition. The organic horizon represents a mixture of processed plant-derived organic matter and soil components. The M horizon, a mineral horizon forming below the O horizon, is characterized by a lower content of organic matter that originates from both decomposition of organic matter and exudation from abundant tree roots (Voríšková et al., 2014; Hartemink et al., 2020). Yang et al. (2017) studied fungal assemblages in two soil profiles using Illumina sequencing datasets and reported that fungal diversity in surface soils was much higher than that in subsurface soils. Soil fungal communities were mainly structured by soil properties, which may be determined by soil horizon rather than soil depth. However, Yang et al. (2017) found that soil properties alone could not sufficiently explain observed variations in the soil fungal communities of the Ngari drylands of the Asiatic Plateau. Variations in soil physicochemical properties between organic and mineral horizons are accompanied by changes in composition of resident fungal diversity and community composition (Du et al., 2017). Thus, it is worth exploring how fungal diversity, community composition, and assembly processes vary in accordance with soil horizons as well as potential principal driving factors.

Dabie Mountain in China is located in the transition zone between the southern warm-temperate and northern subtropical zones, belonging to the humid monsoon climate of the middle and lower reaches of the Yangtze River. The region is characterized by a montane deciduous broad-leaved forest ecology. The main vegetation types are broad-leaved forest or mixed coniferous broad-leaved forest. The area provides a good opportunity to study soil microbial communities due to the presence of its ancient soil strata, complex terrain, high level of vegetation diversity, and abundant rainfall. Although some research has been conducted on fungi around Dabie Mountain, the majority of these studies have focused only on entomogenous fungi or are limited to macrofungi (Wang et al., 2004). Accordingly, we sought to deepen our understanding of fungi in this region and better understand how fungal communities change across soil horizon layers. The specific objectives of the study were to (1) investigate any differences in fungal diversity and community composition between the organic horizon (O horizon) and mineral matter horizon (M horizon) and (2) explore the potential key influencing factors causing any observed differences. We hypothesized that (1) soil horizons mainly drive the variations of soil fungal community composition and diversity and (2) soil organic matter plays a chief role in shaping soil fungal community composition and diversity in microenvironments.

## Materials and Methods

### Study Sites and Soil Sampling

**Figure 1:**
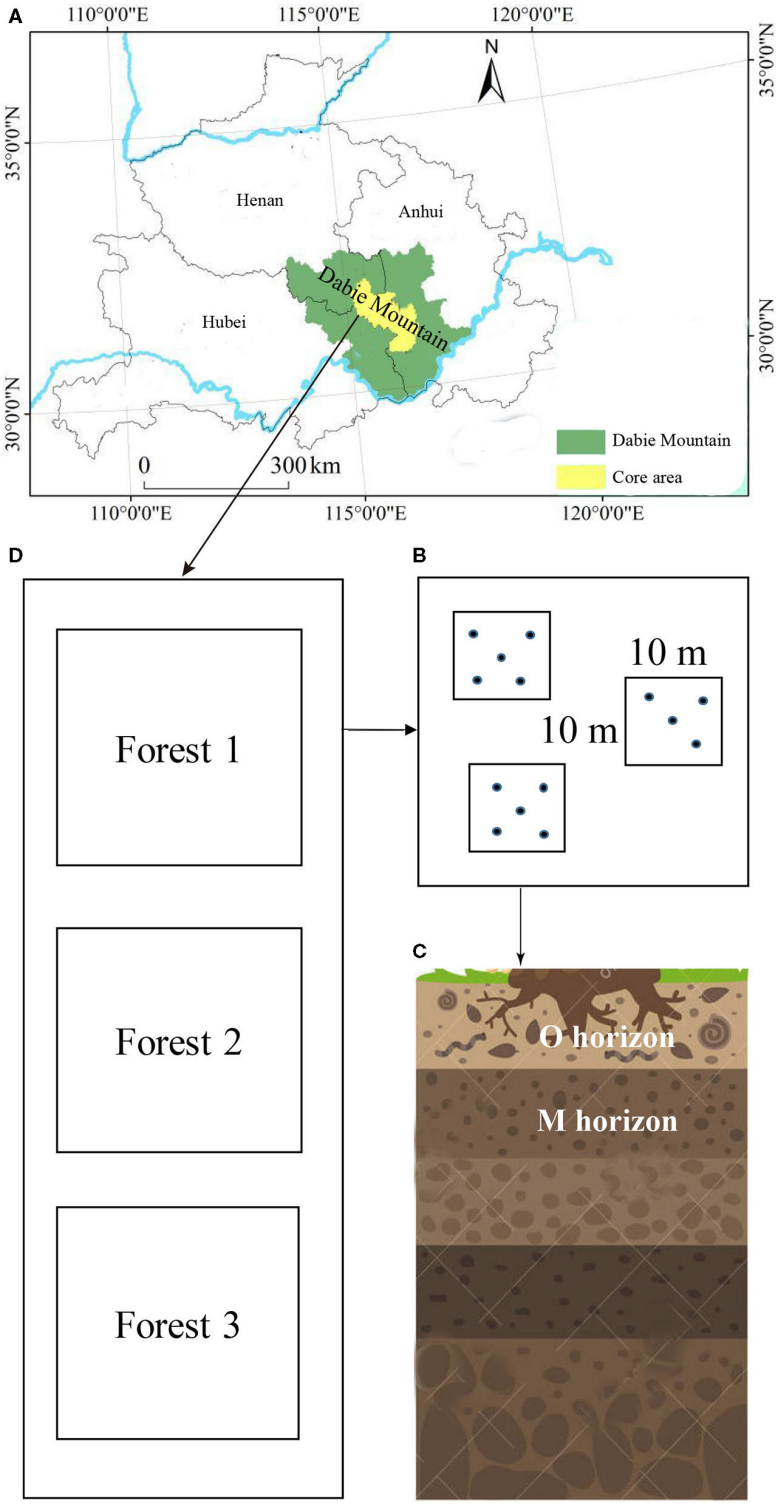
Sampling locations within two sites of Dabie Mountain **(A)** in Anhui Province, China. Subsamples in three subplots **(B)** were collected from the O and M horizon **(C)** of three forest types **(D)**.

Soil samples were collected from the Yaoluoping Nature Reserve (30°57′N−31°06′, 116°02′-116°11′E, elevation 1,076–1,313 m) and Tiantangzhai Nature Reserve (31°10′-31°15′N, 115°38′-115°47′E, elevation 1,113–1,188 m) of Dabie Mountain, Anhui Province, China, in the summer of 2018. Yaoluoping Nature Reserve is ~30 km from Tiantangzhai Nature Reserve. The region is subtropical, with a mean annual precipitation of 1375 mm and a mean annual temperature of 14°C. The main soil type is mountain brown soil. We utilized 12 experimental plots (10 × 10 m in size) in Tiantangzhai Nature Reserve and 10 experimental plots (10 × 10 m in size) in Yaoluoping Nature Reserve across three forest types (about 60 years old) due to the geographical constraints based on GPS coordinates (Figure 1). Each plot was separated from other neighboring plots by at least 100 m. Three identical forest types were selected in the two sites, and the dominant tree species in the three forest types were *Cunninghamia lanceolata* (Lamb.) Hook., *Pinus huangshanensis* Hsia, and a variety of broad-leaved tree species (*Quercus glandulifera* var. *breviptiolata, Castanea seguinii*, and *Q. acutissima*). Details on the environmental factors and soil chemical properties are listed in Supplementary Table 1. The climatic data used in our study were obtained from Yaoluoping and Tiantangzhai weather bureaus.

From each plot, five soil subsamples were taken from the organic horizon (loose and partly decayed organic matter, O horizon) and the M horizon (mineral matter mixed with some humus) using a cylindrical soil borer (Φ 3 cm) and spade. These subsamples were mixed and homogenized. Stones and roots were removed from fresh soil by hand and sieved through a 2-mm mesh. Soil samples were divided into two parts. One part was stored at −80°C for subsequent DNA extraction. The other part was air-dried soil property analysis. In total, 24 soil samples were taken from the Tiantangzhai Nature Reserve and 20 soil samples from the Yaoluoping Nature Reserve.

### Soil Properties

Soil pH was determined in a soil:H_2_O (1:2.5) solution (NY/T1377-2007). Soil available phosphorus (AP) was analyzed using the Bray 1 method (NY/T 1121.7-2014) (Bray and Kurtz, 1945). The high-temperature catalytic combustion method was used to determine the total carbon (TOC), and the Kjeldahl method was used for determining total nitrogen (TON) content of the soil samples (Zhao et al., 2019). The organic carbon content of the soil was determined using the organic carbon–potassium dichromate oxidation spectrophotometric method (HJ615-2011). Soil available potassium (AK) was extracted with ammonium acetate (CH_3_COONH_4_) and measured with a flame photometer (NY/T 889-2004). Alkali-hydrolyzable nitrogen (AHN) was determined by the alkaline hydrolysis diffusion method (LY/T 1229-1999).

### DNA Extraction and Illumina Sequencing

Total DNA from each sample was extracted from 0.2 g of soil using a E.Z.N.A™ Mag-Bind Soil DNA Kit (OMEGA M5635-02) following manufacturer instructions. The fungal internal transcribed spacer 2 (*ITS2*) rDNA region was amplified with the primer pair *ITS3F* (GCATCGATGAAGAACGCAGC) and *ITS4R* (TCCTCCGCTTATTGATATGC) (Nilsson et al., 2019). A two-step PCR was performed for *ITS* amplicon sequencing. The PCR products were purified by Agencourt AMPure XP (MagicPure Size Selection DNA Beads, Transgenic EC401-03). DNA sequences were analyzed using Illumina MiSeq 2 Platform to generate 300 bp paired-end reads. The pooled mixture was purified using a Qubit 3.0 DNA kit (Life Q10212).

### Bioinformatics

Raw reads of the *ITS* region were collected in a MiSeq sequencing machine in fastq format. Primer sequences were modified using Cutadapt V1.10, and then tail region sequences were removed using a slightly lower mass value with Prinseq-lite V0.20.4 (Schmieder and Edwards, 2011). Paired-end reads were merged using Pear V1.9.4 (Zhang et al., 2014). Barcode and primer sequences were cut out using Prinseq-lite V0.20.4, and then N-part sequences, short sequences, and low-complexity sequences (about 200 bp) were removed (Schmieder and Edwards, 2011). Non-specific amplified regional sequences were removed after pretreatment using Usearch V8.1.1831, and then chimera sequences were identified and deleted using Uchime V4.2 (Edgar, 2010). Sequences were checked, and those with less than 80% similarity were eliminated using Blastn. Operational taxonomic units (OTUs) were generated using the Usearch algorithm V8.1.1831 by clustering sequence reads at the 97% similarity threshold (Edgar, 2010). The most abundant sequences were selected for each of the OTUs. Additionally, all singletons were removed during the Usearch clustering process (Edgar, 2010). A total of 1,954,690 sequences were obtained across the soil samples. The sequences ranged from 25,104 to 26,837 high-quality sequences per sample. The final results consisted of 25,044 OTUs in 44 soil samples. Sequencing data sets have been deposited in the NCBI Sequence Read Archive (SRA) database under accession no. PRJNA601887.

### Statistical Analyses

Sequence alignment was analyzed in Unite (http://unite.ut.ee/index.php) and RDP databases (http://rdp.cme.msu.edu/misc/resources.jsp), and those with coverage and similarity below 90% remained unclassified (Wang et al., 2007). The fungal sequence data were rarefied to 25,044 reads per sample. Taxonomy was assigned to fungal OTUs using the RDP classifier (V2.12), based on Bergey's taxonomy, using naïve Bayesian assignment with a mini-confidence of 0.8 which was considered to represent phylum, class, order, family, genus, and species levels. The community compositions of OTUs in the two soil horizons are shown in the VENN figure by using the VennDiagram package in *R* (V3.2). The optimum similarity values of fungal OTUs and taxonomy were selected for statistical analyses based on the relationship between OTU numbers and similarities using Usearch (Edgar, 2010). The beta (β) diversity of fungal OTUs was estimated using Bray–Curtis in the Vegan package of *R*. Hierarchical clustering analysis was used to analyze the relationships between the two soil horizons based on the beta (β) diversity using the Unweighted Pair Group Method with Arithmetic mean (UPGMA) by Bray–Curtis. The rarefaction analysis was analyzed based on OTUs with 90% similarity. The alpha diversity of fungi in each soil horizon was analyzed using the Simpson index, Chao1, and the Shannon index using Mothur V1.30.1 (Schloss et al., 2009). Species richness and community compositions in different soil horizons and their significant differences were analyzed with Stamp (V2.1.3, *P* ≤ 0.05) and visualized in a heatmap using ggplot2 package in *R*. Linear discriminant analysis (LDA) effect sizes (LEfSe) were used to analyze significant differences between the two soil horizons using the Kruskal–Wallis (KW) sum-rank test and (unpaired) Wilcoxon rank-sum test. Principal co-ordinate analysis (PCoA) and nonmetric multidimensional scaling (NMDS) of fungal community compositions were conducted based on Bray–Curtis distance and unweighted UniFrac distance using Vegan package in *R* (Lozupone et al., 2007, 2010). The relationships between fungal communities, samples, and environmental properties were determined using multiple linear regression analysis and redundancy analysis (RDA), which were then used to identify the predominant environmental variables associated with fungal community composition. An analysis of similarities (Anosim) and permutational multivariate analysis of variance (PerMANOVA) were complementary non-parametric analyses based on the Bray–Curtis distance to measure significant differences in community compositions between the two horizons in the vegan package (Yang et al., 2017).

## Results

### Soil Fungal Community Composition in Two Horizons

**Figure 2:**
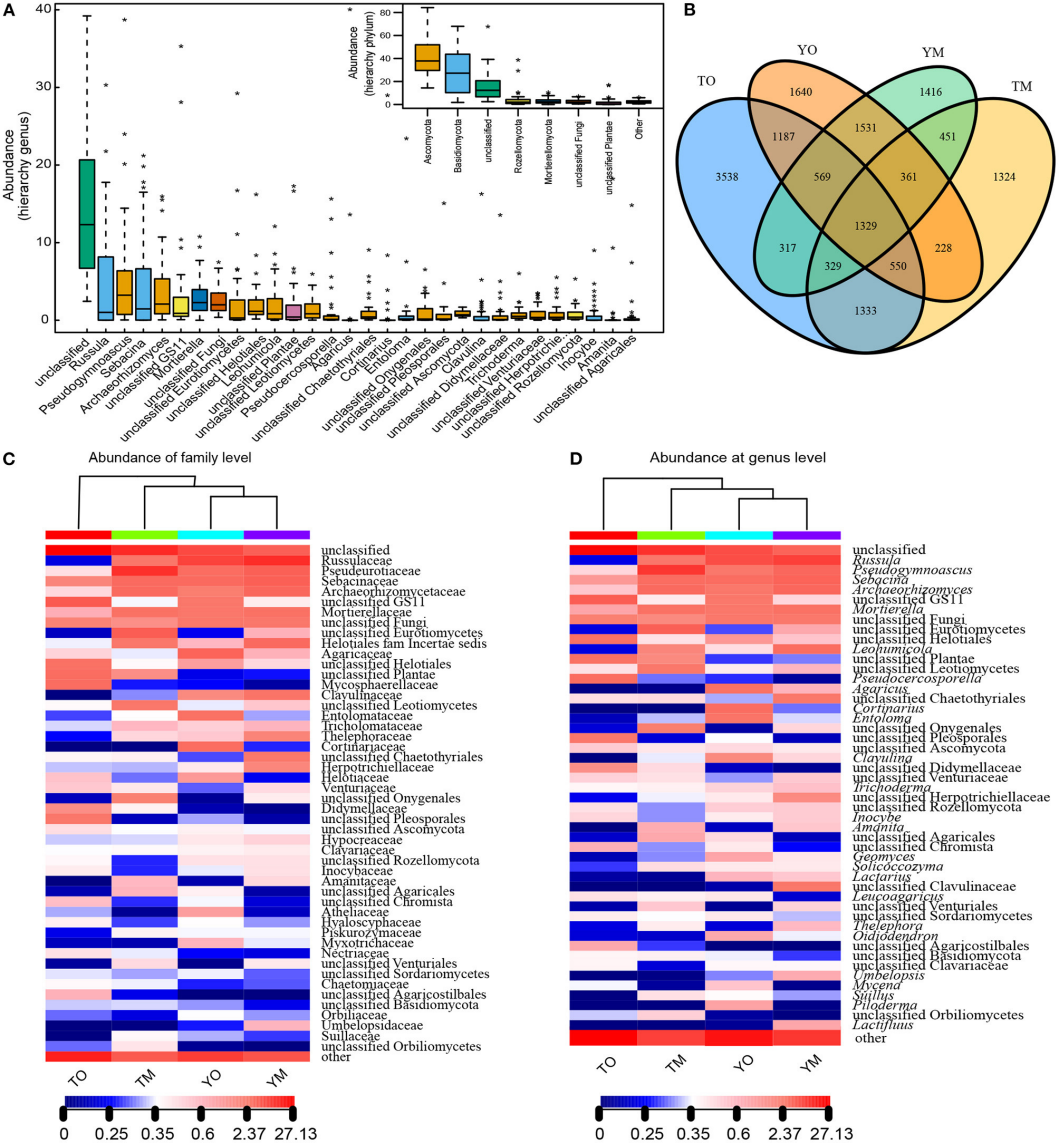
Fungal abundance based on the hierarchy of genus and phylum **(A)** and Venn diagrams of shared and unique OTUs **(B)** in the O and M horizon at Dabie Mountain. UPGMA distance sample cluster tree and the fungal community composition based on family **(C)** and genus **(D)** level in the O and M horizon. TO represents the O horizon at Tiantangzhai Nature Reserve. TM represents the M horizon at Tiantangzhai Nature Reserve. YO represents the O horizon at Yaoluoping Nature Reserve. YM represents the M horizon at Yaoluoping Nature Reserve.

The high-throughput Illumina sequencing yielded a total of 16,103 OTUs from the O and M horizons at 90% sequence identity. The M horizons contained 9,738 OTUs from 967,885 sequences. The dominant fungal phyla across all samples were Ascomycota (36.59%) and Basidiomycota (34.87%), and other and unclassified (23.64%), followed by Rozellomycota (3.42%) and Mortierellomycota (1.48%) (Figure 2A). The O horizon contained 12,912 OTUs from 986,805 sequences (50.48% of all sequences in the filtered dataset) that were dominated by Ascomycota and Basidiomycota (Figure 2A). The dominant genera in the O horizon were *Russula, Pseudocercosporella, Agaricus, Archaeorhizomyces, Cortinarius*, and *Mortierella*. The M horizon contained 9,738 OTUs (49.52% of all sequences in filtered dataset) dominated by Ascomycota, Basidiomycota, and Mortierellomycota (Figures 2B–D). The dominating genera in the M horizon were *Russula, Pseudogymnoascus, Sebacina, Leohumicola, Mortierella*, and *Archaeorhizomyces*. The number of shared OTUs between the two soil horizons was respectively 3,790 and 3,541 at the Yaoluoping Nature Reserve and Tiantangzhai Nature Reserve (Figure 2B).

**Figure 3:**
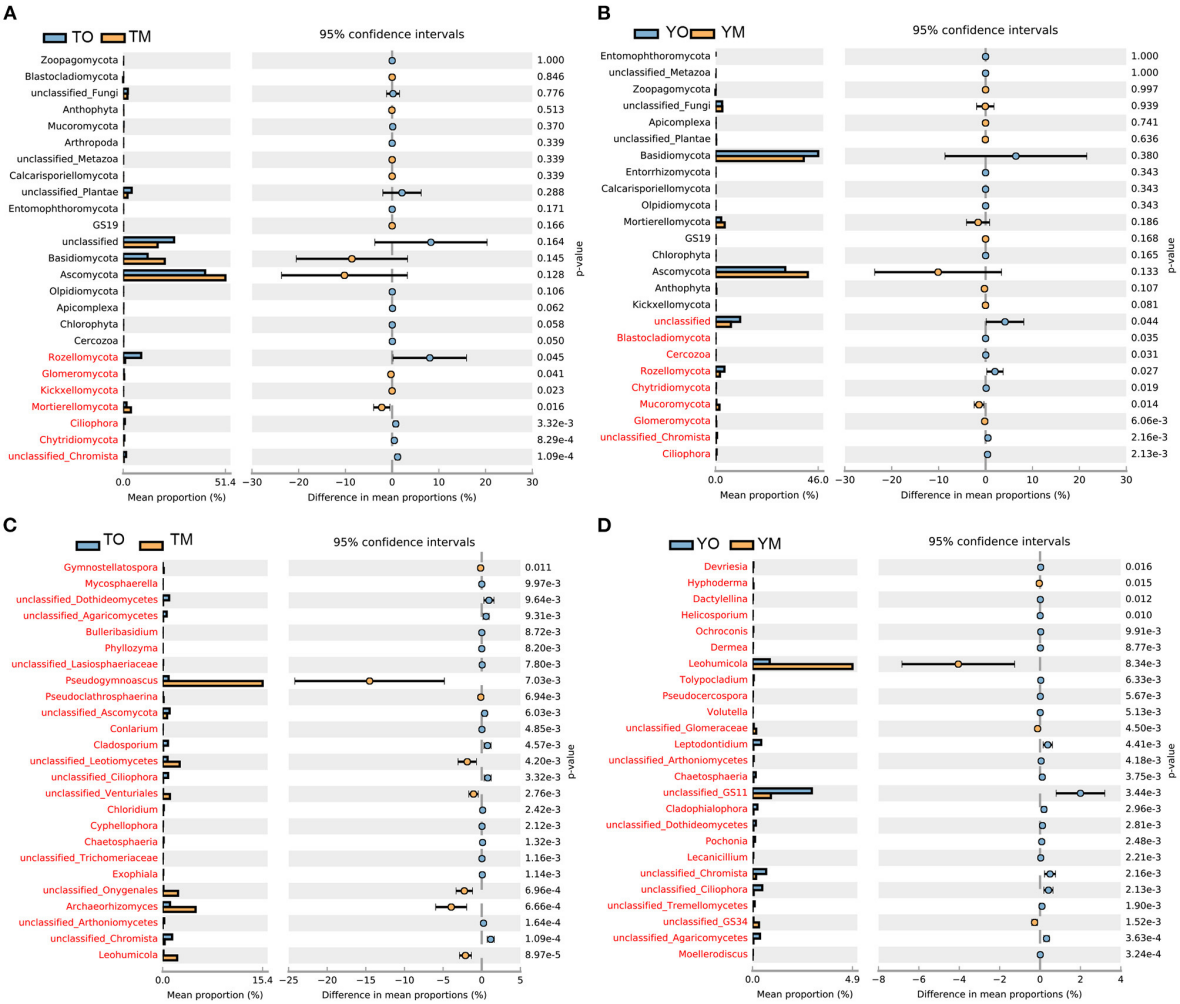
Significantly altered fungal communities between the O and M horizon as measured by the response ratio method at the 95% confidence interval (Welch's *t*-test). Fungal communities of the O and M horizon based on phylum level at Tiantangzhai Nature Reserve **(A)** and Yaoluoping Nature Reserve **(B)**, and fungal communities of the O and M horizon based on genus level at Tiantangzhai Nature Reserve **(C)** and Yaoluoping Nature Reserve **(D)**.

At the phylum levels, there were no significant differences in the abundance of Basidiomycota and Ascomycota between O and M horizons (Figures 3A,B). The abundances of Rozellomycota and Chytridiomycota were significantly higher in the O horizon than that in the M horizon (*P* < 0.05). The abundance of Mortierellomycota in the M horizon was significantly higher than that in the O horizon at the Tiantangzhai Nature Reserve, but there were no significant differences at the Yaoluoping Nature Reserve (*P* < 0.05) (Figures 3A,B). Most fungal genera had a significant difference between the two soil layers (Figures 3C,D). The abundances of *Pseudogymnoascus, Archaeorhizomyces*, and *Leohumicola* were significantly lower in the O horizon than in the M horizon at the Tiantangzhai Nature Reserve (*P* < 0.001) (Figure 3C). The abundance of *Leohumicola* was significantly lower in the O horizon, while *Leptodontidium* and additional unclassified fungi were higher at the Yaoluoping Nature Reserve (*P* < 0.001) (Figure 3D).

**Figure 4:**
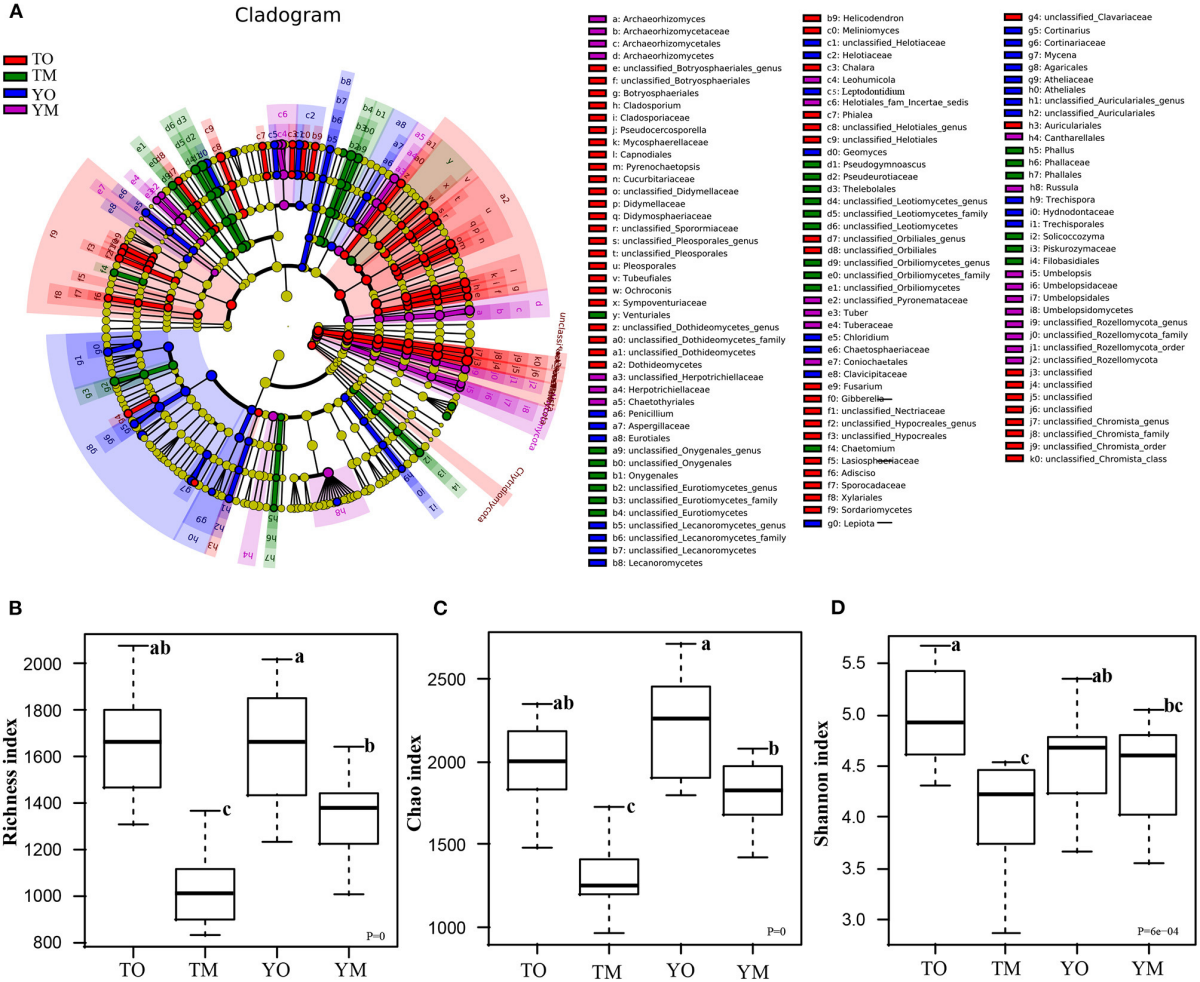
Linear discriminant analysis effect size (LEfSe) among the four groups **(A)**. Effects of soil horizon on fungal richness index **(B)**, Chao1 index **(C)**, and Shannon index **(D)**. TO represents the O horizon at Tiantangzhai Nature Reserve. TM represents the M horizon at Tiantangzhai Nature Reserve. YO represents the O horizon at Yaoluoping Nature Reserve. YM represents the M horizon at Yaoluoping Nature Reserve. The lower case letters mean significant difference.

LDA effect size (LEfSe) analysis revealed different biomarkers in two soil horizons from two sites (Figure 4A). Enrichment of *Dothideomycetes, Pleosporales, Rozellomycota, Capnodiales, Mycosphaerellaceae, Pseudocercosporella, Sordariomycetes, Helotiales*, and *Didymellaceae* were significant in the O horizon at the Tiantangzhai Nature Reserve. Enrichment of *Agaricales, Agaricaceae, Cortinariaceae, Cortinarius, Atheliales, Atheliaceae, Entoloma* sp, and *Helotiaceae* was significant in the O horizon at the Yaoluoping Nature Reserve. Enrichment of *Pseudeurotiaceae, Thelebolales, Pseudogymnoascus roseus, Eurotiomycetes, Onygenales, Leotiomycetesm*, and *Amanitaceae* was significant in the M horizon at the Tiantangzhai Nature Reserve. Enrichment of *Russula, Archaeorhizomycetes, Leohumicola, Archaeorhizomyces, Archaeorhizomycetaceae, Archaeorhizomycetales, Helotiales, Cantharellales, Leohumicola minima, Herpotrichiellaceae, Umbelopsidomycetes*, and *Herpotrichiellaceae* was significant in the M horizon at the Yaoluoping Nature Reserve. Furthermore, we observed significant species enrichment in the two profiles, and biomarkers of the same horizon also differed across locations.

### Soil Fungal Diversity in O and M Horizons

Soil fungal species richness in the O horizon was significantly higher than that in the M horizon (*P* < 0.001), and this depth-based trend was also statistically significant for predicted Chao1 in each site (Figures 4B,C; Table 1). The Shannon index of fungi in the O horizon was significantly higher than that in the M horizon at the Tiantangzhai Nature Reserve (*P* < 0.001), but no significant difference was found at the Yaoluoping Nature Reserve (Figure 4D). The Simpson index showed that there are significant differences between the O and M horizon (Table 1). There was no significant difference between the two sites in fungal species richness and diversity in the O horizon. While species richness and Chao1 were different in the Shannon index of fungi in the M horizon, no significant difference was found between the two sites.

**Table 1:** ANOVA results for the difference of fungal abundance, richness, and diversity between the O horizon and M horizon.

**Samples**	**Abundance**	**Shannon index**	**Simpson**	**Chao1 index**
TM vs. TO	8.5547E-08^**^	0.0001^**^	0.05271	1.0041E-06^**^
YM vs. YO	0.0099^*^	0.5033	0.3573	0.0040^*^
TO vs. YM	0.0029^*^	0.0169^*^	0.6313	0.1696
TM vs. YO	1.3803E-06^**^	0.0229^*^	0.4797	9.2388E-08^**^
TM vs. YM	0.0004^**^	0.1189	0.1514	0.0000^**^
TO vs. YO	0.9142	0.0666	0.1322	0.0667

* *P < 0.05*,

** *P ≤ 0.001*.

*Fungal diversity was expressed as the Shannon index, Simpson and Chao1 index. The bold values mean significant difference*.

**Figure 5:**
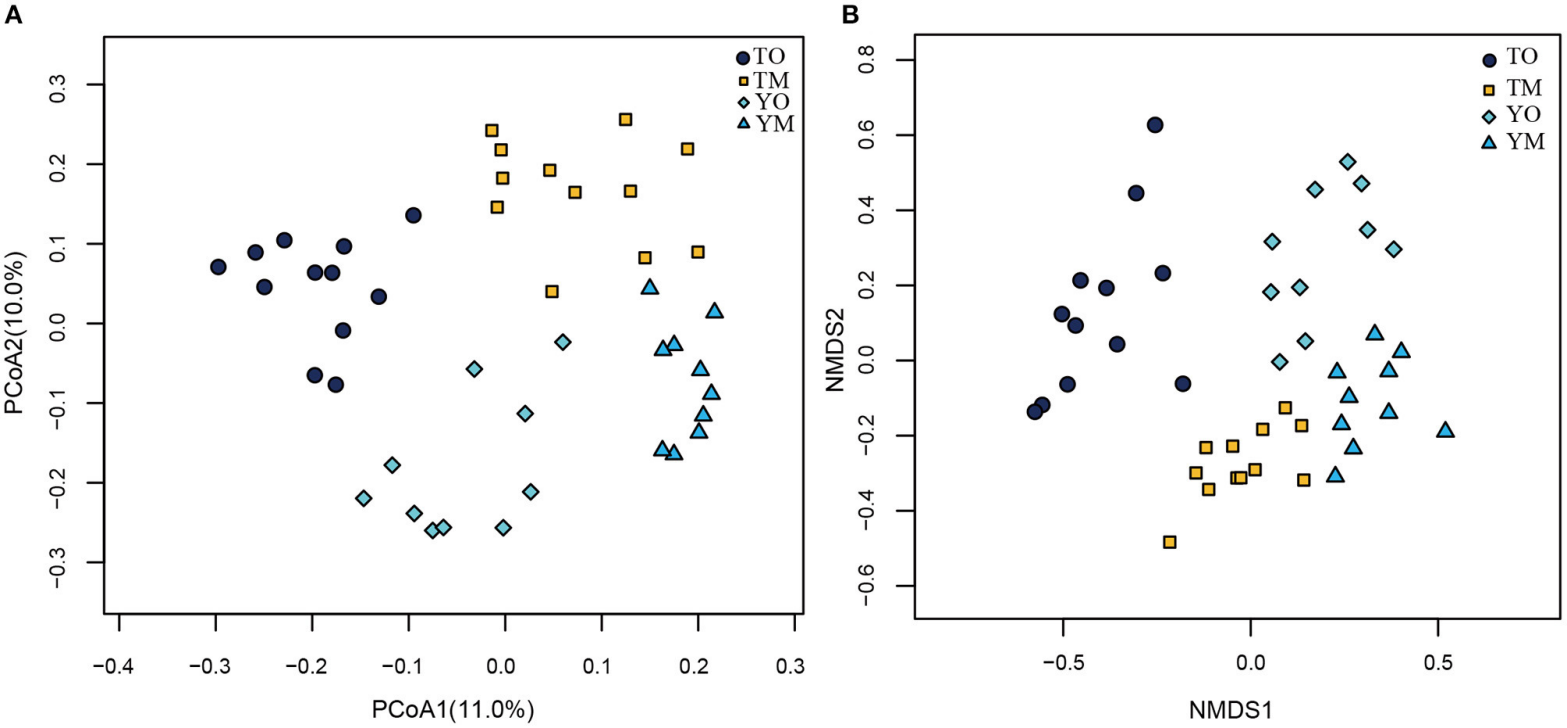
Principal coordinate analysis (PCoA) **(A)** and NMDS plot **(B)** on effect of soil horizon on fungal communities at the O and M soil horizons. TO represents the O horizon at Tiantangzhai Nature Reserve. TM represents the M horizon at Tiantangzhai Nature Reserve. YO represents the O horizon at Yaoluoping Nature Reserve. YM represents the M horizon at Yaoluoping Nature Reserve. TO represents the O horizon at Tiantangzhai Nature Reserve. TM represents the M horizon at Tiantangzhai Nature Reserve. YO represents the O horizon at Yaoluoping Nature Reserve. YM represents the M horizon at Yaoluoping Nature Reserve.

The unweighted PCoA also showed that different soil horizons contained distinct OTUs at each site (Figure 5A). The first two principal components identified by PCoA accounted for 11% (PC1) and 10% (PC2) of overall soil OTUs, respectively. In the visualized two-dimensional NMDS plot, OTUs from the O horizon were very different from the M horizon, and a closer clustering of the points within each soil horizon, despite segregation among two sample sites based on Bray–Curtis distance (Figure 5B). Different soil layers exerted significantly different effects on fungal community structures based on genus, family, and order levels (PERMANOVA and Anosim, *P* < 0.005) (Table 2).

**Table 2:** Differences in fungal community composition between the O and M horizons, based on PERMANOVA (P) in *R* adonis function and Anosim (A).

**Analysis**	**Categories**	**Phylum**		**Class**		**Order**		**Family**		**Genus**	
**P**		**R^**2**^**	***P*-value**	**R^**2**^**	***P*-value**	**R^**2**^**	***P*-value**	**R^**2**^**	***P*-value**	**R^**2**^**	***P*-value**
	TO vs. TM	0.145	0.011^*^	0.276	0.001^**^	0.262	0.001^**^	0.255	0.001^**^	0.253	0.001^**^
	YO vs. YM	0.106	0.122	0.116	0.080	0.135	0.005^*^	0.128	0.001^**^	0.133	**0.002**
	TO vs. YO	0.452	0.001^**^	0.433	0.001^**^	0.278	0.001^**^	0.235	0.001^**^	0.225	0.001^**^
	TM vs. YM	0.289	0.005^*^	0.160	0.007^*^	0.184	0.001^**^	0.179	0.001^**^	0.167	0.002^*^
A		**R**	***P*****-value**	**R**	***P*****-value**	**R**	***P*****-value**	**R**	***P*****-value**	**R**	***P*****-value**
	TO vs. TM	0.237	0.002^*^	0.569	0.001^**^	0.662	0.001^**^	0.734	0.001^**^	0.744	0.001^**^
	YO vs. YM	0.058	0.176	0.116	0.056	0.254	0.004^*^	0.276	0.001^**^	0.325	0.002^*^
	TO vs. YO	0.628	0.001^**^	0.750	0.001^**^	0.707	0.001^**^	0.722	0.001^**^	0.708	0.001^**^
	TM vs. YM	0.099	0.086	0.130	0.043^*^	0.343	0.001^**^	0.379	0.004^*^	0.393	0.002*

* *P < 0.05*,

** *P ≤ 0.001*.

*The bold values mean significant difference*.

### Relative Influences of Soil Properties on Fungal Communities of O and M Horizons

**Figure 6:**
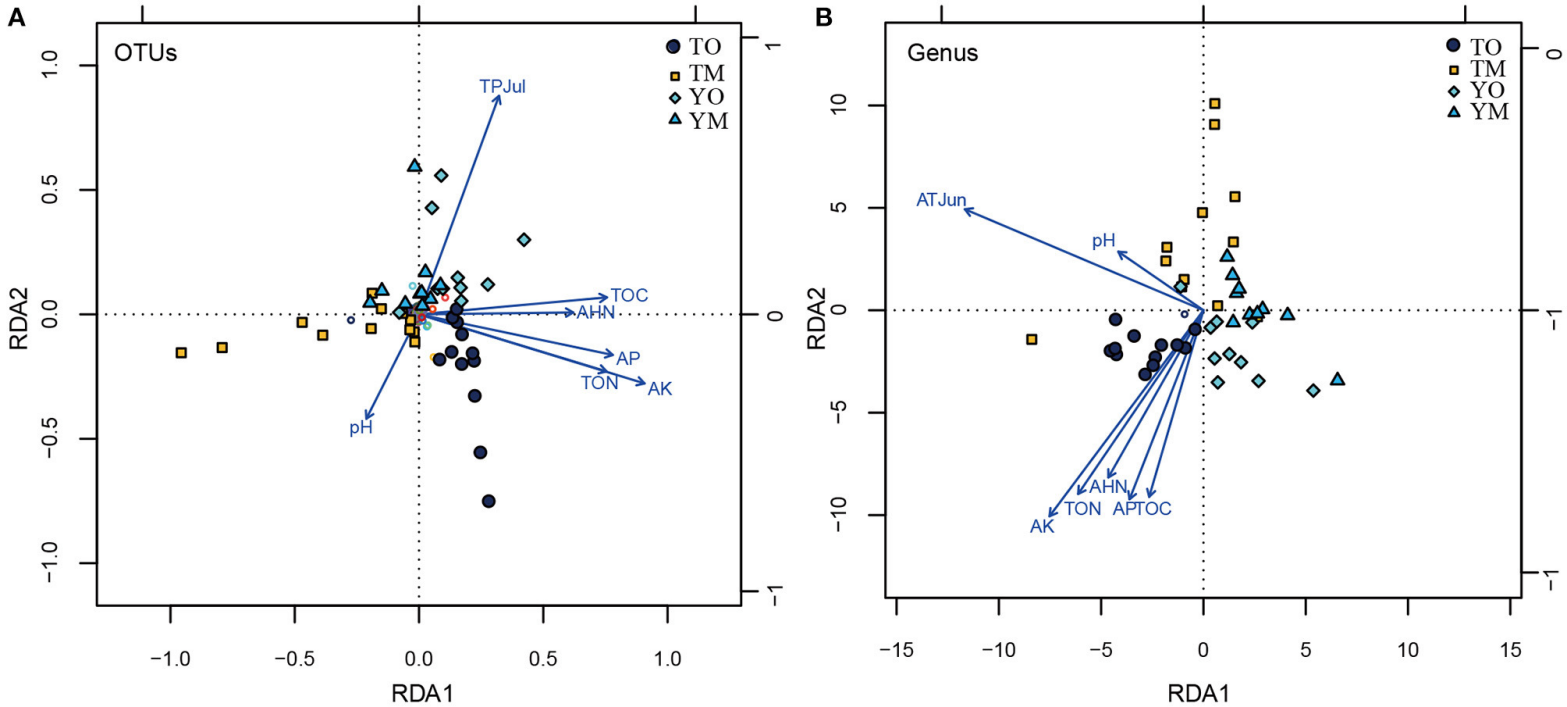
Ordination plots showing the results of the redundancy analysis (RDA) used to explore the relationships between fungal communities and soil properties and climatic variables at the OTU **(A)** and genus **(B)** levels. TOC refers to the total soil carbon, TON refers to the total soil nitrogen, AHN refers to the soil alkali-hydrolyzable nitrogen, AP refers to the available phosphorus, and AK refers to the soil available potassium. TPJul refers to the total precipitation of July at the site. pH refers to soil pH value. TO represents the O horizon at Tiantangzhai Nature Reserve. TM represents the M horizon at Tiantangzhai Nature Reserve. YO represents the O horizon at Yaoluoping Nature Reserve. YM represents the M horizon at Yaoluoping Nature Reserve.

The abundance of OTUs was significantly correlated with the content of TOC, TON, AHN, AK, and AP, while the abundance of OTUs had no significant relationship with pH value and climatic variables (monthly mean temperature, humidity, and total precipitation) (*P* < 0.05). Furthermore, the Chao1 of the OTUs that had no significant relationship with the pH value was significantly correlated with TOC, TON, AHN, AK, AP, air temperature, air humidity, and the total month's precipitation (*P* < 0.05) (Supplementary Table 2). At the OTU level, RDA showed that TOC, TON, AHN, AK, and AP were significantly correlated with the fungal communities of the O horizon (Figure 6A). Moreover, pH negatively associated with the total precipitation of July was a crucial factor to OTU communities in the M horizon, but soil pH had a weak relationship with soil organic matter. At the genus level, the contents of TOC, TON, AHN, AK, and AP were significantly correlated with soil fungal communities in the O horizon, while they had no significant relationship with fungal communities in the M horizon (Figure 6B). Positively correlated pH and total precipitation in July were significant drivers of soil fungal communities in the M horizon at the Tiantangzhai Nature Reserve. However, they had a weak relationship with soil fungal communities at the Yaoluoping Nature Reserve (Figure 6B).

*Archaeorhizomyces, Pseudogymnoascus, Leohumicola*, unclassified Helotiales, unclassified Onygenales of *Ascomycota*, and *Mortierella* of *Mortierellomycota* were significantly correlated with TOC, TON, AHN, AK, and AP content, while they had no significant relationship with pH (*P* < 0.05) (Supplementary Table 2). Certain Basidiomycota genera, such as *Agaricus, Cortinarius*, and *Entoloma*, had no significant relationship with TOC, TON, AHN, AK, and AP content. *Russula* (Basidiomycota) was significantly correlated with precipitation and humidity, while the genus had no significant relationship with soil properties. The genus *Sebacina* was significantly correlated with TON, but had no significant relationship with TOC, AHN, AK, AP, or any of the climatic variables measured. *Pseudocercosporella* of Ascomycota were significantly correlated with pH, AK, and climate conditions.

## Discussion

Although it is known that soil fungal diversity and composition vary across soil profiles (Guo et al., 2019; Zhang et al., 2019), unexplained variation in fungal communities across soil horizons can be attributed to the relationship between soil microenvironment and fungi. In this study, we investigated the effect of soil horizons on fungal communities in two sites. Our results provide solid evidence that soil horizons significantly affect fungal communities. Furthermore, fungal diversity and species richness were significantly higher in the organic horizon than in the M horizon, which was consistent with the findings of other studies (van Leeuwen et al., 2017; Yang et al., 2017). The contents of TOC, TON, AHN, AK, and AP in the O horizon were higher than those in the M horizon, which was in accordance with the results of Du et al. (2017). Fungal community composition, diversity, and abundance had significant relationships with soil organic matter. We speculate that nutritional trends in the soil profile, even small changes, can cause significant differences in fungal communities (Wakelin et al., 2016). Moreover, certain genera belonging to Ascomycota, such as *Archaeorhizomyces, Pseudogymnoascus, Leohumicola*, unclassified Helotiales, unclassified Onygenales, and *Mortierella* significantly correlated with soil organic matter. Soil organic matter for these genera was a dominant factor in the regulation of the soil fungal community composition and affected the establishment of fungal communities (Zhang et al., 2019). Our results support the theory that soil nutrients affect microbial diversity and microbial composition (Du et al., 2017). Soil organic matter has been shown to be a crucial driver for fungal communities in topsoil (Tian et al., 2017; van Leeuwen et al., 2017). The effects of nutritional differentiation on fungi can explain the variance between fungal structures in the two soil horizons. However, it was difficult to discern the effects of individual factors on fungal community composition and diversity as well as understand how factors affect fungal communities.

There was a more significant difference between the two soil layers than between the two sites, and no obvious variations in soil fungal communities were found between different sites in the O horizon. Our results agreed with other studies (Taylor et al., 2014; Yang et al., 2017) in which the soil horizon partitioning of fungal communities was shown at a regional scale alongside the uniform community composition of surface soil. Our results strongly suggest that changes in soil organic matter between the two soil horizons determined soil fungal communities rather than geographic distance. Some studies showed that forest type drives the variation of soil fungal communities (Ren et al., 2019; Sheng et al., 2019). In forest, tree species identity which largely determines litter resources can affect microenvironmental conditions by influencing soil nutrient content (Xiao et al., 2019). Forest type had significant effects on chemical properties, biological properties and nutrient cycles, but microbial diversity indices did not significantly differ among forest types (Kooch and Bayranvand, 2017; Nakayama et al., 2019). In the present study, we speculate that the similar fungal communities between the two sites may be due to similar forest types. However, we note that our study did not consider the effect of tree species community composition in each forest type on fungal communities.

Soil pH could be a good predictor of fungal community structure (Liu et al., 2018). However, we found that soil pH did not play a significant role in shaping the fungal communities observed in our study, such as *Entoloma, Russula, Cortinarius, Agaricus, Archaeorhizomyces*, and *Leohumicola*, which had no significant relationship with soil pH. A possible reason could be the adaption of fungal species to broad variations in soil pH (Rousk et al., 2010). Moreover, we speculate that ectomycorrhizal fungi, such as *Entoloma, Russula*, and *Cortinarius*, were mainly affected by host plant, whereas saprophytic fungi, such as *Agaricus, Archaeorhizomyces*, and *Leohumicola*, were more likely to be influenced by soil organic matter rather than soil pH. In this study, total monthly precipitation before sampling significantly affected soil fungal communities, especially for fungal communities from the M horizon, and our results suggest that precipitation plays an important role in determining fungal community structure, in agreement with past studies (Sheng et al., 2019). Water is the primary driver in ecological processes and significantly influences plant and microbial species diversity (Bell et al., 2009). Water is crucially needed for soil organic matter absorption and metabolism activities.

Ascomycota and Basidiomycota were the dominant phyla in the O and M horizons in the forest ecosystems of our study region (He et al., 2017; Wang et al., 2017; Liu et al., 2018). The dominance of these phyla in soils might be related to their ability to degrade complex lignocellulose components, close involvement in root exudation assimilation, and symbiotic relationships with plant roots in forest soils (Liu et al., 2018; Zhang et al., 2019). However, the abundances of *Rozellomycota* and *Chytridiomycota* in the O horizon were significantly higher than those in the M horizon. This may be because some species of the two phyla can be saprophytic and pathogenic on the residual limbs of plants and animals and also decompose their remains in topsoil (Zhang et al., 2019); moreover, Rozellomycota obtain nutrients by invading plants (Cai et al., 2011). Some species of Mortierellomycota, such as *Mortierella horticola, M. zonata*, and *M. parvispora* which are mycorrhizal or pathogenic forest fungi, were more abundant in the M horizon than in the O horizon. At the genus level, our results showed that *Russula* and *Mortierella* were the most dominant groups in the two soil horizons. Ectomycorrhizal fungi such as *Russula* (*R. californiensis, R. cyanoxantha, R. foetens, R. subvinosa*) usually forms symbiotic relationships with species of *Pinus* or *Quercus*. *Mortierella*, such as *M. humilis*, is known to be found in higher abundance in healthy soils (Gams et al., 1972). Previously, Tedersoo et al. (2020) proposed that root associations with mycorrhizal fungi benefit plants by enhancing their nutrient access and stress tolerance as well as mediate plant interaction with other soil microbes, such as helping plants obtain organic carbon from soil horizons through mycorrhizal networks (Kong et al., 2016). In this study, we speculate that soil horizons together with plants affect soil fungal communities.

Different fungal communities were detected in different soil horizons, similar to the observations of Voríšková et al. (2014). These authors found that *Mycena, Sistotrema*, and *Cryptoccocus* were the most abundant genera in the litter horizon, while *Russula* and *Lactarius* were enriched in the deeper horizons. Du et al. (2017) found that the abundance of *Inocybe, Paxillus*, and *Agaricales* showed marked differences with the soil profile. The community composition and abundances of soil fungi were found to vary between O and M horizons. This is likely because the amount of soil organic matter could influence fungal community composition (Sayer and Tanner, 2010; Guo et al., 2019). Moreover, based on the fungal groups present in each horizon, we speculate that fungi feeding on cellulose and lignin tend to occur in the O horizon (Zhang et al., 2019).

## Conclusions

We explored changes in soil fungal communities and soil organic matter in both the O and M horizons at two sites around Dabie Mountain. Our results show that differences between the soil horizons significantly affect the fungal community structure at various taxonomical levels. Soil organic matter, along with TOC, TON, AHN, AK, and AP contents, is positively associated with fungal community composition, abundance, and diversity, so that a more prolific fungal community occurs in the O horizon. Climatic variables have only a slight effect on the fungal communities.

## Data Availability Statement

The datasets presented in this study can be found in online repositories. The names of the repository/repositories and accession number(s) can be found below: https://www.ncbi.nlm.nih.gov/PRJNA601887.

## Author Contributions

XL collected soil samples, analyzed the dataset, and wrote the manuscript. KL collected the soil samples and analyzed some dataset. YS and GY collected soil and then analyzed the soil properties. WY analyzed processed some figures. PM and HG modified the manuscript. All authors contributed to the article and approved the submitted version.

## Conflict of Interest

The authors declare that the research was conducted in the absence of any commercial or financial relationships that could be construed as a potential conflict of interest.
