# Supplementary material for: Fungal Community Composition and Diversity Vary With Soil Horizons in a Subtropical Forest
Source: Front Microbiol. 2021 Jul 1;12:650440. doi: 10.3389/fmicb.2021.650440 (PMC8281208; doi:10.3389/fmicb.2021.650440)
Supplement: Supplementary file 1 [file Data_Sheet_1.docx]

**Table S1** Summary of soil organic matters and climate conditions at Yaoluoping Nature Reserve and Tiantangzhai Nature Reserve of Dabie Mountain.

| **Parameters** | **TO** | **TM** | **YO** | **TM** |
| --- | --- | --- | --- | --- |
| Total carbon (TOC)（mg/kg） | 110891.67±47187.79 | 105691.67±4797.30 | 100450.0±49652.86 | 88808.33±7512.63 |
| Total nitrogen (TON)（mg/kg） | 8483.67±1871.06 | 4507.25±400.64 | 8040.30±1989.76 | 2885.70±512.80 |
| pH value | 5.00±0.46 | 5.00±0.14 | 4.65±0.22 | 4.90±0.12 |
| Alkali-hydrolyzable nitrogen (AHN) (mg/kg） | 589.08±136.73 | 381.00±57.31 | 647.30±135.65 | 247.41±74.96 |
| Available potassium (AK) (mg/kg） | 335.75±74.68 | 171.50±46.75 | 301.40±79.02 | 111.10±16.26 |
| Available phosphorus (AP) (mg/kg） | 13.52±5.96 | 3.33±1.01 | 13.01±5.48 | 2.39±0.61 |
| Monthly mean temperature in June (ATJun) (℃） | 23.0 | | 18.9 | |
| Monthly mean temperature in July (ATJul) (℃） | 26.7 | | 22.5 | |
| Monthly mean temperature in August (ATAug) (℃） | 25.4 | | 21.4 | |
| Relative humidity in June (ARHJun) (%) | 80 | | 89 | |
| Relative humidity in July (ARHJul) (%) | 82 | | 91 | |
| Relative humidity in August (ARHAug) (%) | 86 | | 92 | |
| Total precipitation in June (TPJun) (mm) | 114.2 | | 120.5 | |
| Total precipitation in July (TPJul) (mm) | 225.3 | | 247.2 | |
| Total precipitation in August (TPAug) (mm) | 235.2 | | 290.8 | |
| Slope | 15—22° | | 15—22° | |
| Elevation | 1026—1176 | | 1065—1312 | |
| Slope aspect | Southeast—Southwest | | Southeast—Southwest | |

TO represent O horizon at Tiantangzhai Nature Reserve. TM represent A horizon at Tiantangzhai Nature Reserve. YO represent O horizon at Yaoluoping Nature Reserve. YM represent A horizon at Yaoluoping Nature Reserve.

**Table S2** The effect of soil organic matters and climate conditions on fungal communities based on OTUs, genus and family level based on Pearson Correlation Coefficient. Some factors that didn’t significantly affect fungal communities, are not shown.

| **Classified unit** | **TOC** | **TON** | **pH** | **AHN** | **AK** | **AP** | **ATJun** | **TPJul** |
| --- | --- | --- | --- | --- | --- | --- | --- | --- |
| OTU num | **0.0000**** | **0.0001**** | 0.2513 | **0.0006*** | **0.0000**** | **0.0000**** | 0.2244 | 0.2244 |
| Chao1 index | **0.0001**** | **0.0013*** | 0.1486 | **0.0037*** | **0.0007**** | **0.0002**** | **0.0178*** | **0.0178*** |
| **Genus** |  |  |  |  |  |  |  |  |
| *Agaricus* | 0.6894 | 0.9183 | 0.3034 | 0.4354 | 0.7103 | 0.6314 | 0.1631 | 0.1631 |
| *Archaeorhizomyces* | **0.0248*** | **0.0056*** | 0.7384 | **0.0064*** | **0.0241*** | **0.0101*** | 0.3948 | 0.3948 |
| *Cortinarius* | 0.1149 | 0.1021 | 0.1805 | 0.0625 | 0.1795 | 0.1475 | 0.0953 | 0.0953 |
| *Entoloma* | 0.6911 | 0.7514 | 0.6551 | 0.2602 | 0.8573 | 0.6919 | 0.1253 | 0.1253 |
| *Leohumicola* | **0.0047*** | **0.0002**** | 0.825 | **0.0027*** | **0.0001**** | **0.001**** | **0.0346*** | **0.0346*** |
| *Mortierella* | **0.0066*** | **0.0064*** | 0.5002 | **0.023*** | **0.0462*** | **0.0085*** | 0.4697 | 0.4697 |
| *Pseudocercosporella* | 0.7104 | 0.1869 | **0.0013*** | 0.3928 | **0.0037*** | 0.4702 | **0.026*** | **0.026*** |
| *Pseudogymnoascus* | **0.0226*** | **0.021*** | 0.6248 | **0.0496*** | **0.0023*** | **0.0215*** | 0.2082 | 0.2082 |
| *Russula* | 0.9272 | 0.3368 | 0.3737 | 0.515 | 0.356 | 0.6791 | **0.0007**** | **0.0007**** |
| *Sebacina* | 0.0931 | **0.0274*** | 0.6509 | 0.0522 | **0.0085*** | 0.358 | 0.2299 | 0.2299 |
| Unclassified Chaetothyriales | 0.3388 | 0.0714 | 0.4922 | 0.1222 | 0.0948 | **0.038*** | 0.3026 | 0.3026 |
| Unclassified Onygenales | **0.0054*** | **0.0134*** | 0.184 | **0.0358*** | **0.0056*** | 0.8715 | 0.0797 | 0.0797 |
| Unclassified Pleosporales | 0.5271 | **0.0013*** | 0.291 | **0.0107*** | **0.0085*** | 0.358 | 0.1586 | 0.1586 |
| **Family** |  |  |  |  |  |  |  |  |
| Russulaceae | 0.9514 | 0.2237 | 0.2794 | 0.3851 | 0.2360 | 0.5578 | **0.0001* *** | **0.0001* *** |
| Pseudeurotiaceae | **0.0260*** | **0.0219*** | 0.7549 | 0.0606 | **0.0026*** | **0.0294*** | 0.3549 | 0.3549 |
| Sebacinaceae | 0.0808 | **0.0246*** | 0.6574 | **0.0471*** | 0.1635 | 0.0524 | 0.2107 | 0.2107 |
| Archaeorhizomycetaceae | **0.0248*** | **0.0056*** | 0.7384 | **0.0064*** | **0.0241*** | **0.0101*** | 0.3948 | 0.3948 |
| Unclassified GS11 | 0.2207 | **0.0425*** | 0.8522 | 0.1184 | 0.0993 | **0.0295*** | 0.2338 | 0.2338 |
| Mortierellaceae | **0.0064**** | **0.0064*** | 0.4908 | **0.0227*** | **0.0467*** | **0.0083*** | 0.4777 | 0.4777 |
| Unclassified Fungi | 0.1461 | 0.3246 | 0.1932 | 0.4080 | 0.4881 | 0.2979 | 0.1664 | 0.1664 |
| Unclassified Eurotiomycetes | **0.0364*** | 0.0946 | 0.2166 | 0.1348 | 0.0948 | **0.0380*** | 0.0750 | 0.0750 |
| Helotiales fam Incertae sedis | **0.0112*** | **0.0006**** | 0.6552 | **0.0071*** | **0.0002**** | **0.0038*** | **0.0219*** | **0.0219*** |
| Agaricaceae | 0.1280 | 0.3083 | 0.1658 | 0.1893 | 0.7245 | 0.1253 | 0.1231 | 0.1231 |
| Unclassified Helotiales | **0.0002**** | **0.0017*** | **0.0028*** | **0.0119*** | 0.0905 | **0.0053*** | 0.2530 | 0.2530 |
| Mycosphaerellaceae | 0.6997 | 0.1792 | **0.0012*** | 0.3781 | **0.0033*** | 0.4554 | **0.0253*** | **0.0253*** |
| Clavulinaceae | 0.8322 | 0.5179 | 0.3480 | 0.4409 | 0.5443 | 0.6215 | 0.0612 | 0.0612 |
| Unclassified Leotiomycetes | **0.0054*** | **0.0100*** | 0.6279 | **0.0185*** | **0.0044*** | **0.0049*** | 0.1120 | 0.1120 |
| Entolomataceae | 0.7004 | 0.7391 | 0.7284 | 0.2642 | 0.8747 | 0.7000 | 0.1672 | 0.1672 |
| Tricholomataceae | 0.4233 | 0.3937 | 0.8141 | 0.4163 | 0.4204 | 0.5028 | 0.7334 | 0.7334 |
| Thelephoraceae | 0.2289 | 0.0676 | 0.3170 | 0.1522 | 0.2026 | 0.0764 | **0.0478*** | **0.0478*** |
| Cortinariaceae | 0.0964 | 0.0918 | 0.1552 | 0.0562 | 0.1610 | 0.1250 | 0.0834 | 0.0834 |

*P* <0.05 *, *P*≤0.001 **

TO represent O horizon at Tiantangzhai Nature Reserve. TM represent A horizon at Tiantangzhai Nature Reserve. YO represent O horizon at Yaoluoping Nature Reserve. YM represent A horizon at Yaoluoping Nature Reserve. TOC refers to soil total carbon, TON refers to soil total nitrogen, AHN refers to soil alkali-hydrolyzable nitrogen. AP refers to available phosphorus. AK refers to soil available potassium. TPJul refers to total precipitation of July. pH refers to soil pH value. ATJun refers to average temperature in June.
